# Bioprinting and Intellectual Property: Challenges, Opportunities, and the Road Ahead

**DOI:** 10.3390/bioengineering12010076

**Published:** 2025-01-15

**Authors:** Antreas Kantaros, Theodore Ganetsos, Florian Ion Tiberiu Petrescu, Elli Alysandratou

**Affiliations:** 1 Department of Industrial Design and Production Engineering, University of West Attica, 12244 Athens, Greece; 2 “Theory of Mechanisms and Robots” Department, Faculty of Industrial Engineering and Robotics, Bucharest Polytechnic University, 060042 Bucharest, Romania; florian.petrescu@upb.ro; 3 School of Humanities, Hellenic Open University, 26335 Patras, Greece

**Keywords:** bioprinting, intellectual property, bioinks, bioprinting hardware, digital design files, ethical considerations, patentability, regulatory frameworks, personalized medicine, regenerative medicine

## Abstract

Bioprinting, an innovative combination of biotechnology and additive manufacturing, has emerged as a transformative technology in healthcare, enabling the fabrication of functional tissues, organs, and patient-specific implants. The implementation of the aforementioned, however, introduces unique intellectual property (IP) challenges that extend beyond conventional biotechnology. The study explores three critical areas of concern: IP protection for bioprinting hardware and bioinks, ownership and ethical management of digital files derived from biological data, and the implications of commercializing bioprinted tissues and organs. Employing a multidisciplinary approach, the paper analyzes existing IP frameworks, highlights their limitations when applied to bioprinting, and examines ethical dilemmas, such as ownership of bioprinted human tissues and the commodification of biological innovations. Findings suggest that current IP laws inadequately address the complexities of bioprinting, particularly in managing the intersection of proprietary technologies and ethical considerations. The study underscores the need for adaptive legal and ethical frameworks to balance innovation with equitable access and sustainability. Recommendations include the development of tailored IP policies for bioprinting and enhanced international collaboration to harmonize legal protections across jurisdictions. This work aims to provide a comprehensive foundation for stakeholders to navigate the rapidly evolving landscape of bioprinting IP.

## 1. Introduction

Additive manufacturing, or 3D printing, constitutes a groundbreaking method of production that has the capacity to profoundly alter numerous industries. Fundamentally, 3D printing is the layer-by-layer construction of three-dimensional objects from digital models, employing a diverse array of materials, including plastics, metals, ceramics, and even biological substances [1]. This technology has significantly evolved since its introduction in the 1980s, transforming from a specialized tool for rapid prototyping into a mainstream manufacturing process that facilitates the creation of complex geometries that would be challenging, if not impossible, to achieve with conventional subtractive manufacturing techniques [2,3].

The disruptive potential of 3D printing spans multiple sectors, including aerospace, automotive, healthcare, fashion, and construction, among others. In the aerospace sector, 3D printing enables the fabrication of lightweight components that improve fuel efficiency and minimize waste, as it permits the design of intricate structures that optimize strength-to-weight ratios [4,5]. In the automotive sector, manufacturers are increasingly utilizing additive manufacturing for both prototype and the production of customized components, thereby reducing lead times and costs associated with conventional manufacturing methods [6,7,8].

Bioprinting, an emerging technology that integrates additive manufacturing with biological sciences, is positioned at the forefront of transformative innovations in healthcare and biomedical research [9]. This process entails the precise, layer-by-layer deposition of bioinks—comprising living cells, biomaterials, and other biologically relevant substances utilized to fabricate complex, three-dimensional tissue structures that emulate the morphology and functionality of native biological tissues [10]. The technology has gained substantial prominence due to its potential to address critical challenges in medicine, including organ scarcity, tissue regeneration, and the development of personalized therapeutic strategies [11]. Bioprinting’s capacity to directly print living cells onto scaffolds and fabricate functional tissues has offered new capabilities in regenerative medicine, drug development, and in vitro disease modeling, thereby significantly altering the sector of medical research and treatment.

Bioprinting, as a distinct subset of biotechnology, differs significantly from other technologies utilizing human cells, such as CAR T cell therapy. While CAR T cell technology focuses on modifying immune cells for therapeutic purposes, bioprinting employs additive manufacturing techniques to fabricate complex, three-dimensional bio-logical structures, including tissues and organs. This process integrates living cells, biomaterials, and bioactive substances in precise spatial arrangements to mimic the morphology and functionality of native tissues. The interdisciplinary nature of bioprinting, which combines expertise from engineering, materials science, and cell biology, presents unique challenges in determining ownership and patentability of innovations. Additionally, the layered and modular construction of bioprinted products introduces complexities that extend beyond the scope of traditional IP frameworks, necessitating nuanced approaches to address the interplay of biological and techno-logical components [12].

The scope of bioprinting’s impact extends beyond healthcare into multiple domains, including pharmaceutical research, diagnostics, and industries such as cosmetics and food. In the pharmaceutical sector, bioprinted tissues are being explored for their utility in drug discovery and toxicity testing, offering more accurate, human-relevant models than traditional in vitro systems [13]. Moreover, bioprinted organs and tissues are being investigated for their potential application in transplantation, providing a viable solution to the global organ shortage crisis [14]. However, despite its remarkable potential, bioprinting introduces a host of intellectual property (IP) challenges that require careful consideration. The rapid pace of technological advancement, coupled with the inherent complexity of bioprinted products, necessitates the development of robust IP frameworks to safeguard innovation while addressing ethical concerns and ensuring compliance with regulatory standards [15]. The interdisciplinary nature of bioprinting—spanning 3D printing, materials science, cell biology, and engineering—can complicate the determination of inventorship when contributions from diverse fields overlap or are interdependent. While private law contracts provide mechanisms for assigning ownership, they may not fully address the nuanced challenges posed by the convergence of biological and technological innovations in bioprinting.

In addition, intellectual property (IP) plays a critical role in fostering innovation in biotechnology, providing legal protection to inventors and ensuring that the fruits of their labor can be monetized and commercially exploited. In the biotechnology sector, IP covers a wide range of inventions, including genetic materials, biopharmaceuticals, diagnostic methods, and medical devices [16]. Traditional forms of IP protection, such as patents, copyrights, and trade secrets, are essential for securing the rights to novel inventions and preventing unauthorized use or replication. However, the rapidly advancing nature of biotechnologies, such as gene editing and tissue engineering, presents unique challenges in defining the scope of IP rights, especially in the context of biological materials and processes that may be inherently complex and not easily reduced to traditional patentable inventions [17]. This complexity becomes even more pronounced in emerging fields like bioprinting, where using advanced technology with living organisms introduces unique legal challenges. While existing patent laws, such as those under the European Patent Convention (EPC), provide a robust and flexible framework, targeted clarifications addressing the unique convergence of biological materials and engineering processes in bioprinting could better accommodate the field’s ethical and multidisciplinary complexities.

Bioprinting, as a new subset of biotechnology, introduces unique IP challenges that are not easily addressed by existing frameworks. The use of living cells in bioprinted products complicates ownership and patentability, particularly when considering issues related to the modification, reproduction, or commercialization of biological materials [18]. Bioprinted tissues and organs, for instance, raise fundamental questions about the ownership of the biological components (such as human cells) incorporated into the final product as well as the methods used to manipulate and print them. Furthermore, the highly interdisciplinary nature of bioprinting—combining expertise in 3D printing, materials science, cell biology, and engineering—poses challenges in determining the rightful ownership of inventions that may involve multiple contributors or innovative processes that span across various technological domains [19]. These complexities necessitate the development of tailored IP policies and legal frameworks to address the evolving nature of bioprinting and to balance the protection of innovation with the ethical considerations inherent in working with living organisms.

**Figure 1:**
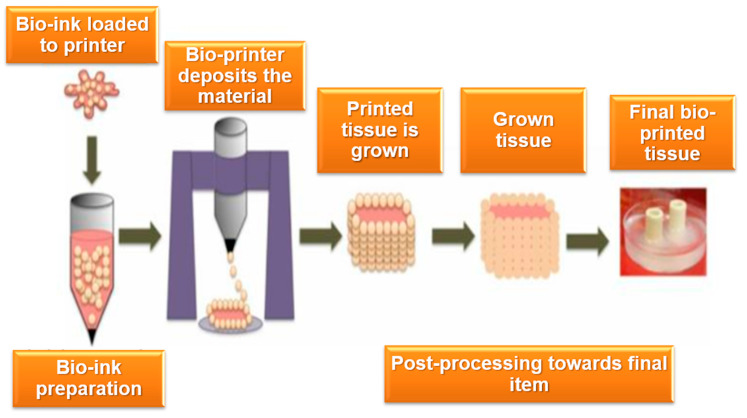
Key stages of the bio-printing workflow.

The scope of intellectual property issues in bioprinting encompasses several distinct but interconnected areas, each with its own set of legal and ethical considerations. One of the primary areas involves the bioprinting hardware and materials used in the production of biological structures [20]. The development of specialized 3D printers capable of handling bioinks, along with the innovation of biocompatible materials and scaffolds, is a key component of bioprinting technology. IP protection for these hardware and material innovations often takes the form of patents, which safeguard novel technologies and manufacturing processes. This area also extends to the development of bioinks composed of living cells, growth factors, and other bioactive components, where proprietary rights may be claimed for new formulations and methods of synthesis [21]. Figure 1 depicts the distinct stages of a bioprinting process.

Another critical area in bioprinting IP pertains to the digital files used to design and construct bioprinted structures. These files, which contain precise biological data derived from imaging techniques such as MRI or CT scans, are essential for accurately replicating biological tissues and organs [22]. The digital design files themselves may be subject to copyright protection, and in some cases, patents may apply to the innovative methods used to generate or optimize these files for bioprinting applications. Furthermore, the commercialization of bioprinted products introduces additional IP challenges, particularly as bioprinted tissues and organs approach clinical applications. Ownership and licensing issues arise when bioprinted products are developed for commercial purposes, such as bioprinted skin for burn victims, customized drug testing models, or even organ transplants [23]. Here, considerations about patenting, trade secrets, and the potential for regulatory hurdles must be navigated to ensure that bioprinted products can be brought to market while respecting IP rights and adhering to ethical standards. Table 1 addresses the aforementioned data.

Thus, the objective of this paper is to critically examine the intellectual property (IP) challenges specific to the field of bioprinting, with a focus on the technological, legal, and ethical complexities that arise as this technology advances. Bioprinting, which combines 3D printing with biological sciences to create living tissues and organs, poses unique IP challenges that extend beyond the scope of conventional biotechnology. These challenges include determining the ownership and patentability of bioinks, the digital files representing biological data, and the bioprinting processes themselves. As the technology evolves, traditional IP frameworks may struggle to provide adequate protection for the wide array of innovations involved in bioprinting, necessitating a re-evaluation of existing laws to accommodate these novel developments. The paper seeks to explore how these IP issues impact both research and commercialization efforts, offering a deeper understanding of the legal obstacles that may hinder the progress of bioprinting technologies. By considering various aspects of bioprinting, such as the use of living cells and the commercialization of bioprinted products, the paper will evaluate how IP laws can be adapted to manage the complex interplay of technological development, bioethics, and public policy. The objective is not only to provide insights into how the bioprinting industry can navigate these challenges but also to contribute to the broader discourse on how IP protection should evolve in response to the rapidly changing landscape of biotechnological advancements. Ultimately, this work seeks to propose strategies that protect innovators’ rights while addressing the ethical implications of working with living organisms and ensuring that the benefits of bioprinting can be realized in a fair and sustainable manner.

## 2. Intellectual Property in Bioprinting Hardware and Materials

Bioprinting hardware consists of specialized components designed to handle the unique challenges of printing living tissues and biological structures. At the core of bioprinting technology are the bioprinters themselves, which operate on similar principles to traditional 3D printers but incorporate additional features to manage biological materials [24]. These printers use precision mechanisms to deposit bioinks layer by layer, utilizing various printing methods, such as extrusion, inkjet, and laser-assisted bioprinting. Bioprinters are equipped with multiple print heads or nozzles to facilitate the simultaneous deposition of different bioinks, allowing for the creation of complex multi-material structures. The printer’s precision and control are critical for ensuring that cells are accurately placed within a scaffold, preserving their viability and ensuring the proper alignment necessary for tissue development [25]. Additionally, bioprinting machines often incorporate temperature and humidity controls to maintain the optimal environment for the bioinks during printing, which adds further complexity to the bioprinter’s design [26].

Bioinks and support materials are integral to the functionality of bioprinting, as they provide the biological foundation for tissue creation. Bioinks are typically composed of living cells, extracellular matrices, and biomaterials that are designed to mimic the natural environment of human tissues [27,28]. These inks must be formulated to support cell viability and promote tissue growth, requiring advanced material science techniques [29]. Additionally, support materials are often used during the printing process to maintain structural integrity, especially for more complex, multi-layered tissue constructs. These materials, which may be biodegradable or dissolvable, are printed alongside the bioinks and later removed once the tissue has stabilized [30]. The development of novel bioinks and support materials—ranging from hydrogels to synthetic biomaterials—represents a significant area of innovation within the bioprinting field. As such, the unique combination of bioprinter mechanisms, bioinks, and support materials presents numerous opportunities for patentable inventions but also raises critical IP issues related to the protection of proprietary formulations, printing techniques, and hardware designs [31,32].

In the bioprinting field, patent law plays a crucial role in protecting innovations related to the hardware components of bioprinting equipment, such as the printers themselves, print heads, and other specialized mechanisms designed to handle biological materials [33]. Patents can be granted for novel inventions that demonstrate an inventive step and are useful in the manufacturing process of bioprinted materials. For instance, bioprinter mechanisms, such as those responsible for the precision placement of cells or the control of the printing environment (temperature, humidity, etc.), may be patented if they introduce new technological features that enhance the printing process or improve the quality of bioprinted tissues [34]. The patenting of such hardware innovations encourages further technological advancement, as it provides inventors with exclusive rights to their inventions for a set period, thereby promoting investment in research and development. However, the complexity of bioprinting hardware may lead to disputes over the novelty and patentability of incremental improvements, as bioprinting is an interdisciplinary field involving advances from 3D printing, robotics, and biotechnology.

Proprietary materials, particularly bioinks, also present significant IP challenges in bioprinting [35]. Bioinks are critical to the success of bioprinting technologies, as they must not only support the structural integrity of printed tissues but also ensure cell viability, growth, and differentiation. As such, bioinks may consist of living cells, extracellular matrices, growth factors, and other biomaterials, each of which may have unique compositions or formulations. Patenting bioinks is a complex process, as it requires demonstrating the novelty and utility of a particular composition or method of creating the ink [36]. Innovations in bioink formulations, such as those that improve cell encapsulation or optimize cell proliferation, can be patented as long as they meet the criteria of novelty and non-obviousness. However, the patenting of biological materials, especially when they involve living organisms or human-derived cells, raises unique ethical and legal concerns, particularly in relation to ownership and access to these materials [37]. The commercialization of bioinks further complicates IP issues, as companies must navigate patent protection for both the materials themselves and the methods used to create them.

In addition to patenting bioinks, material IP extends to the methods of production, manipulation, and delivery of these materials. Patent protection may cover the process of synthesizing bioinks, such as novel techniques for incorporating cells into hydrogels or developing biodegradable scaffolds that promote tissue regeneration [38]. These methods may also be patentable if they offer a more efficient or effective approach than existing technologies. However, the interdisciplinary nature of bioprinting, which blends biotechnology, chemistry, and materials science, poses challenges in determining the rightful ownership of innovations. For example, the question of whether an improvement to a bioink’s formulation or a method for creating a more efficient bioprinting process is patentable can lead to legal disputes, particularly if multiple parties are involved in the development of different components of the bioprinting system [39]. As the field continues to evolve, patenting strategies will need to address these complexities, ensuring that both hardware and material innovations are adequately protected while fostering collaboration and further development in bioprinting technologies.

These challenges necessitate the development of hybrid intellectual property frameworks that reconcile the principles of open access with adequate protections for innovation. Such frameworks could involve licensing mechanisms that allow for collaborative development while retaining safeguards for the intellectual contributions of individual stakeholders. For example, open-source licenses tailored to bioprinting could specify conditions under which shared designs or formulations can be used, modified, or commercialized, ensuring that contributors receive appropriate recognition and benefits [40]. Moreover, fostering an environment of trust among open-source collaborators is essential for addressing these IP concerns. Trust can be facilitated by implementing clear guidelines for crediting contributions, establishing transparent governance structures for opensource projects, and creating incentives that align with the ethos of open access while providing tangible benefits for innovators [41].

In this context, the field of bioprinting has seen relatively few legal cases specifically addressing hardware or material-related intellectual property disputes. However, relevant precedents can be drawn from broader 3D printing and biotechnology industries, which often intersect with bioprinting technologies.

One notable case involves Amgen Inc. (Thousand Oaks, CA, USA) v. Sanofi (Paris, France) (2019), which highlighted the complexities of patenting biological innovations [42]. The dispute revolved around the sufficiency of patent descriptions, emphasizing the need for clear and detailed claims when seeking protection for biological materials. This principle applies equally to bioinks, where the specificity of formulations or the methods for embedding living cells into scaffolds can be contested [43].

In terms of hardware, parallels can be drawn with cases such as Stratasys (Rehovot, Israel) v. Microboards Technology (Chanhassen, MN, USA) (2015), which focused on the infringement of 3D printing technologies [44,45]. While not specific to bioprinting, the decision underscored the importance of safeguarding novel mechanisms in printer designs, including precision controls and multi-material deposition technologies [46]. As bioprinting incorporates even more specialized hardware, disputes over incremental improvements and overlapping patents are likely to emerge.

Additionally, the patenting of bioinks and bioprinting processes raises ethical and legal challenges akin to those in the Association for Molecular Pathology v. Myriad Genetics, Inc. (Salt Lake City, UT, USA) (2013) case, which addressed the patentability of naturally occurring genetic sequences. Similarly, questions of whether biological elements within bio-inks—such as human-derived cells or growth factors—can be patented continue to pose unresolved dilemmas [47,48,49]. These precedents offer a foundation for understanding how courts may address future bioprinting disputes, though the specificities of the field will require new legal interpretations.

Open-source practices have begun to influence bioprinting, particularly in academic and non-profit research environments, where collaboration is key to advancing the field. Open-source platforms, such as the NIH 3D Print Exchange or initiatives like Open Bioprinting, aim to democratize access to bioprinting technologies by sharing designs for hardware, software, and sometimes even bioink formulations [50,51].

While open-source practices foster innovation by allowing researchers and developers to build upon shared knowledge, they also complicate the traditional IP landscape. Licensing models, such as Creative Commons or GNU General Public Licenses (GPL), are often adapted to bioprinting to ensure shared contributions remain accessible while recognizing contributors’ efforts [52]. However, these licenses may lack the specificity needed to address the unique challenges of bioprinting, such as ensuring the ethical use of bioink formulations containing human cells.

One prominent example is the use of open-source bioprinters, such as the INKREDIBLE by CELLINK, whose design philosophy blends open collaboration with proprietary components [53]. This hybrid approach highlights the tension between protecting IP and fostering community-driven innovation. While some groups advocate for entirely open designs to accelerate discovery, others argue that proprietary elements are necessary to sustain commercial viability and fund further research [54].

To address these tensions, the bioprinting community may benefit from tailored open-source licenses that explicitly define permissible uses of shared designs, particularly when bioinks or scaffolding materials are involved. These licenses could include clauses on non-commercial use, attribution requirements, or ethical guidelines for working with human-derived materials, ensuring that the open-source movement supports both innovation and accountability. Table 2, depicts the key challenges, legal complexities, and proposed solutions for intellectual property in bioprinting hardware and materials.

Ultimately, addressing these challenges is critical for realizing the full potential of open-source bioprinting. Balancing the advantages of open collaboration with the need to protect intellectual assets ensures an environment where innovation can thrive without undermining the communal exchange of knowledge and resources. By harmonizing these priorities, the open-source movement can continue to drive transformative advancements in bioprinting, expanding its impact across fields such as personalized medicine, drug discovery, and regenerative therapies

## 3. Digital Files and Biological Data in Bioprinting

Digital files play a pivotal role in bioprinting, serving as the backbone for the fabrication of tissues and organs. These files, typically designed using bio-CAD (computer-aided design) software, encode critical information about the geometry, cellular organization, and material composition of the constructs [55]. Acting as digital blueprints, bioCAD files are often derived from patient-specific data, such as CT or MRI scans, allowing for the customization of biological constructs to meet precise clinical needs. This level of detail not only facilitates the production of highly accurate tissue models but also enables iterative design processes, where modifications can be simulated and optimized before physical printing.

The intersection of these digital files with intellectual property (IP) protections highlights a complex legal and ethical landscape. On one hand, bio-CAD files represent intellectual achievements, often embedding years of research, innovative design techniques, and proprietary methodologies. These aspects align them closely with copyright protections, which safeguard original creative works. The visual and structural designs contained within these files may be eligible for copyright, shielding them from unauthorized reproduction [56]. However, the functional aspects of bio-CAD files, such as specific arrangements of cells or encoded instructions for bioprinting machinery, often fall outside the scope of copyright, being more aligned with patent or trade secret protections.

Patents provide another layer of protection for bio-CAD files, particularly when they encode patented bioprinting processes, methods, or compositions [57]. For instance, a digital file that incorporates a novel method for arranging stem cells within a scaffold or specifies the use of proprietary bioinks may directly extend the scope of a patent. In such cases, unauthorized use of the file could constitute patent infringement, even if the file itself is not explicitly patented. Trade secret laws also play a critical role, as bio-CAD files often encapsulate confidential and proprietary knowledge. Maintaining the secrecy of these files through robust digital security measures becomes paramount, especially in an era in which cyber-threats and unauthorized access pose significant risks to proprietary data [58].

The use of biological data in bioprinting, particularly when creating digital models derived from patient-specific genetic or anatomical data, raises significant ethical, legal, and privacy concerns. These digital models, often based on data obtained through imaging technologies such as CT or MRI scans or genetic sequencing, are essential for tailoring bioprinted tissues and organs to meet individual patient needs. However, the sensitive nature of the underlying biological data presents challenges in terms of ownership, consent, and data protection.

Regarding patentability of computer-implemented inventions in bioprinting, in both the United States and Europe, the patentability of computer-implemented inventions (CII) has become increasingly relevant in fields like bioprinting, where digital files and biological data are essential components of the process. These inventions, which may include software, algorithms, or methods implemented by a computer, are assessed under specific legal frameworks.

In the United States, computer-implemented inventions are typically patentable if they satisfy the fundamental criteria of patentability: novelty, non-obviousness, and utility. However, the U.S. has established an additional test under the “Alice decision”, which excludes abstract ideas from being patentable unless they involve an inventive concept that transforms the abstract idea into a patent-eligible application. In the context of bioprinting, this could apply to digital methods of designing bioinks or algorithms for optimizing the 3D printing process, provided they meet the required standards for patentability.

In Europe, the European Patent Office (EPO) assesses computer-implemented inventions under specific guidelines. According to the EPO’s Guidelines for Examination (2024), a computer-implemented invention may be patentable if it provides a technical solution to a technical problem. This requirement ensures that the invention is more than just an abstract idea. Specifically, the invention must involve a technical contribution to the state of the art, which could include, for example, methods for controlling 3D printers through software or algorithms used for processing biological data to create bioinks.

For bioprinting, the software tools that enable the design, customization, or optimization of bioinks and 3D printed tissues are prime candidates for CII patenting. The EPO’s Guidelines (F-IV, 3.9) [59] emphasize that the mere use of a computer does not make an invention technical unless the computer is performing a task that would be considered a technical contribution. Similarly, the Guidelines (G-II, 3.6) [60] note that the specific requirements for sufficiency of disclosure for CII can involve explaining the underlying technical processes in sufficient detail to enable reproducibility.

**Figure 2:**
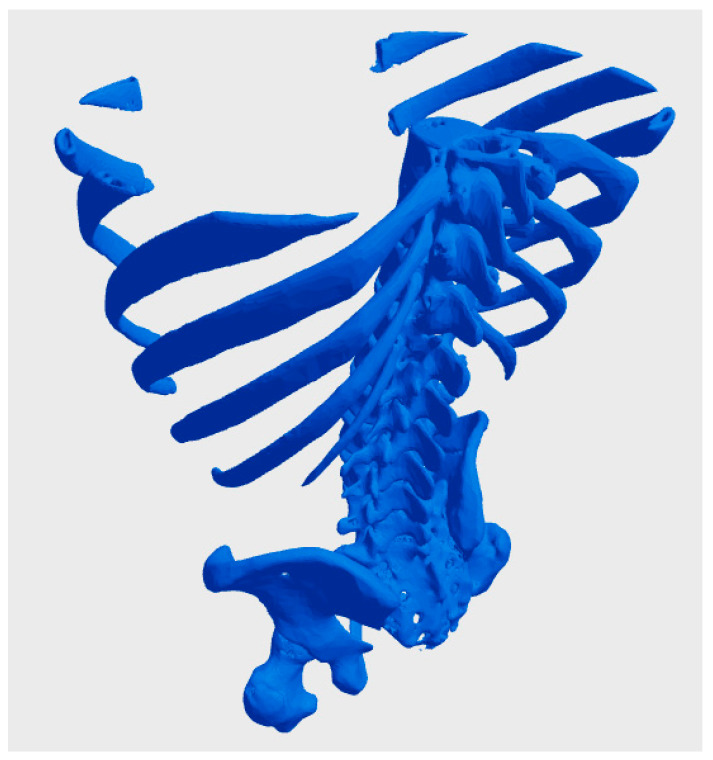
bio-CAD model derived from a real CT scan of a patient’s thorax and pelvis, freely available on the Thingiverse website under a Creative Commons license [61].

A central issue in using biological data for bioprinting is determining who owns the digital models derived from a patient’s biological or genetic information. In many jurisdictions, individuals retain ownership of their biological data, including genetic sequences and imaging results, as an extension of their right to bodily autonomy [60]. However, once this data is processed into a digital model, questions arise about whether the healthcare provider, the bioprinting company, or the individual retains rights over the derived product. For instance, a hospital that facilitates data collection might claim partial ownership of the resulting digital model, particularly if it contributes to the processing or uses proprietary software. Similarly, companies that develop specialized algorithms or tools to convert raw biological data into bio-CAD files might assert intellectual property claims over the models, potentially diminishing the patient’s control over their use. Figure 2 illustrates a bio-CAD model derived from a real CT scan of a patient’s thorax and pelvis, freely available on the Thingiverse website under a Creative Commons license. This model exemplifies key challenges in the context of bioprinting, particularly those related to the ownership and ethical considerations of patient data. The accessibility of this data, while promoting open-source collaboration, also raises important questions regarding privacy, consent, and the appropriate use of personal medical information in digital and 3D printing applications [61].

Privacy concerns further complicate the use of biological data in bioprinting. Patient-specific files contain highly sensitive information that, if misused or disclosed without consent, could lead to ethical violations or discrimination. Genetic data, in particular, carry risks of misuse, such as predictive analysis for insurance or employment purposes. To mitigate these risks, bioprinting practitioners must comply with stringent data protection laws such as the EU’s General Data Protection Regulation (GDPR) or the U.S. Health Insurance Portability and Accountability Act (HIPAA) [62,63]. These frameworks mandate informed consent for data collection, require anonymization to protect patient identities, and enforce strict security protocols to safeguard stored data.

Moreover, the global nature of bioprinting research raises cross-border issues concerning the use of biological data. Data collected in one jurisdiction may be processed or used in another, leading to conflicts between differing privacy and owner-ship laws. For example, genetic data collected in a country with strong data protection laws may not receive the same level of protection if transferred to a country with weaker regulatory frameworks. This creates a need for international harmonization of policies governing the use of biological data in bioprinting to ensure consistent ethical standards and legal protections. Consent mechanisms also play a critical role in addressing ownership and privacy concerns. Patients must be fully informed about how their biological data will be used, including the potential for creating digital models, the scope of data sharing, and any commercial implications. Dynamic consent models, which allow patients to adjust their preferences over time, offer a promising approach to managing ongoing consent in bioprinting applications.

Finally, the ethical dimensions of using biological data must be considered. Beyond legal compliance, bioprinting stakeholders have a moral obligation to respect the autonomy and dignity of individuals whose data is used. This includes ensuring that patients retain meaningful control over their data, receive adequate information about its potential uses, and benefit equitably from advancements derived from their contributions. The licensing and sharing of biodata files in bioprinting research and clinical applications necessitate a careful balance between promoting innovation and protecting sensitive information [64]. These files, which may include patient-derived genetic sequences, anatomical models, or structural designs for tissues and organs, often require explicit agreements to delineate rights and responsibilities among stakeholders. Licensing mechanisms serve as a legal tool to govern the use, distribution, and modification of these files, establishing parameters that align with both ethical principles and regulatory requirements [65].

The use of open licenses in research environments fosters collaborative innovation, enabling researchers to access and build upon shared data to advance the field. However, such openness must be tempered with safeguards to prevent the misuse of sensitive information. Licensing agreements must specify conditions under which the data may be used, such as prohibiting commercial exploitation or requiring ethical compliance for derivative works [66]. In clinical settings, proprietary licenses are more common, reflecting the need to protect patient confidentiality and the commercial value of specialized data-processing algorithms or proprietary bio-CAD files. These agreements often include non-disclosure clauses, emphasizing the importance of restricting access to authorized personnel or entities.

The ethical considerations surrounding data sharing in bioprinting are grounded in principles of consent, equity, and accountability. Patients or donors of biological data must be fully informed about how their contributions will be used, stored, and shared [67]. Consent forms should address the scope of data usage, including whether files may be distributed to third parties or modified for derivative works. Dynamic consent models, which allow individuals to periodically update their permissions, offer a practical approach to managing consent in longitudinal studies or ongoing collaborations. Equity is also critical; licensing frameworks should ensure that contributors and under represented groups benefit proportionally from the advancements made possible by shared data. Revenue-sharing agreements, free access to resultant technologies, or collaborative capacity-building initiatives can help achieve this goal.

The modification of biodata files introduces further complexities, particularly in determining ownership of derivative works. Licensing agreements must clarify whether modified files are subject to the same terms as the originals or if new licenses may apply. The creation of derivative works, such as altered genetic sequences or restructured anatomical models, raises ethical questions about the extent to which modifications can be considered independent innovations versus extensions of the original contribution. Additionally, ensuring the integrity and traceability of modified files is critical to maintaining accountability within collaborative networks.

To address these challenges, the bioprinting community must develop frameworks that integrate ethical considerations with robust legal protections. Blockchain technology offers potential solutions by providing transparent and immutable records of data transactions, facilitating accountability while protecting sensitive information. Similarly, collaborative governance structures involving diverse stakeholders can help balance competing interests and ensure that licensing agreements promote both innovation and fairness.

Technological solutions offer additional mechanisms for protecting bio-digital files, addressing challenges of ownership, traceability, and unauthorized distribution. Digital rights management (DRM) systems can restrict access to files, controlling who can view, edit, or distribute them [68]. While effective for enforcing permissions, DRM must be carefully designed to avoid impeding legitimate research activities or stifling innovation.

Watermarking is another promising approach, embedding identifiable markers within biodata files to establish authorship and detect unauthorized modifications [69]. Watermarks can serve as evidence in disputes over data misuse, providing a tamper-evident trail that ensures accountability. For instance, bio-CAD files used in collaborative bioprinting projects could be watermarked with unique identifiers linked to the originating institution or contributor [70].

Blockchain technology presents a more advanced solution, offering decentralized and immutable records of data transactions. By leveraging blockchain, institutions can create transparent systems for tracking the sharing and modification of bio-digital files [71]. Blockchain enables smart contracts, which automate licensing agreements and enforce conditions such as time-limited access or restrictions on derivative works. This approach enhances trust and accountability, particularly in international collaborations, where regulatory compliance can vary [72]. Table 3, depicts the intellectual property challenges, legal complexities, and proposed solutions for digital files and biological data in bioprinting.

The integration of these technological solutions into licensing and data-sharing practices must align with ethical principles, ensuring that the interests of all stakeholders are protected. By leveraging innovative tools such as DRM, watermarking, and blockchain alongside robust legal and ethical frameworks, the bioprinting community can safeguard sensitive data while promoting collaborative progress. This balanced approach will enable the responsible sharing and modification of biodata files, driving advancements in both research and clinical applications.

## 4. IP Challenges in Bioprinted Tissues and Organ Replication

Bioprinted tissues and products, particularly those designed for medical applications, present unique intellectual property (IP) challenges. As these innovations often involve proprietary techniques, materials, and designs, questions about the ownership, protection, and replication of bioprinted tissues are becoming increasingly significant [73]. For instance, companies and researchers invest heavily in developing methods to arrange cells in specific structures that mimic natural tissues or improve functionality. These proprietary arrangements, encoded in bio-CAD files or derived through patented processes, are critical assets, yet their replication raises complex IP concerns.

One significant issue involves the enforceability of patents on bioprinted tissues. While processes and materials used in bioprinting, such as bioinks or printing method-ologies, are generally patentable, the tissues themselves may fall into a legal grey area. If bioprinted tissues closely replicate naturally occurring structures, they may not meet the novelty requirement under patent law [74]. However, tissues that incorporate enhancements—such as synthetic scaffolds, unique cellular arrangements, or functional modifications—are more likely to be eligible for patent protection. The challenge lies in distinguishing between what constitutes an unpatentable “product of nature” and an innovation that qualifies for IP rights [75]. Moreover, as bioprinting technology evolves, the ease with which proprietary tissues can be replicated by third parties using the same or slightly modified processes increases the risk of IP infringement.

**Figure 3:**
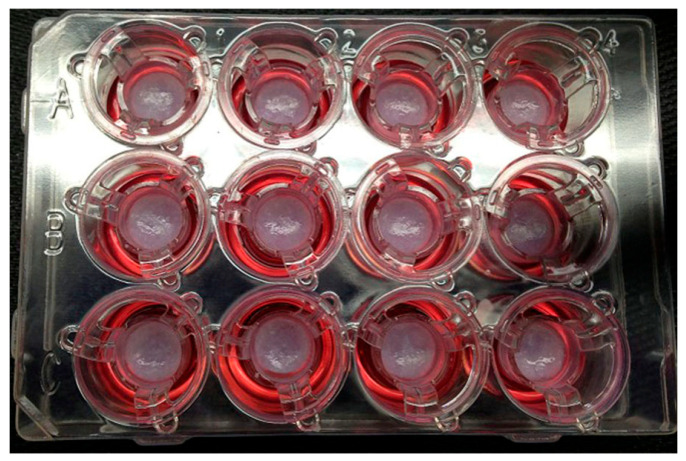
Pictures of 3D bioprinted tissues in a 12-well transwell plate showing reproducible tissue shape from well to well [76].

Ethical and legal concerns further complicate the landscape, especially in the context of bioprinted human tissues for personalized medical applications [75]. Ownership of bioprinted tissues raises profound ethical questions. If a tissue is derived from a patient’s cells and designed specifically for their use, should the patient retain any ownership rights over the bioprinted product? While current legal frameworks typically grant ownership to the entity that created the product, such as a bioprinting company or research institution, this approach may conflict with patients’ moral claims to their biological material. For instance, the patient’s contribution of cells or genetic data is integral to the bioprinting process, making it ethically contentious to exclude them from ownership discussions. Figure 3 depicts pictures of 3D bioprinted tissues in a 12-well transwell plate showing reproducible tissue shape from well to well [76].

Additionally, the commercialization of bioprinted tissues introduces concerns about accessibility and equity. High costs associated with proprietary bioprinting technologies and products may limit their availability to wealthy individuals or well-funded healthcare systems, exacerbating existing disparities in medical care. Licensing agreements for bioprinted tissues must therefore consider not only the rights of creators but also the broader societal implications of restricting access to life-saving technologies [77].

Another critical aspect is the potential for exploitation of vulnerable populations. For example, individuals in resource-limited settings may feel compelled to donate biological materials for research or commercial purposes, only to see the resulting bioprinted products patented and sold without any benefit to their communities. Ethical guidelines and legal safeguards must be in place to ensure that contributions are fairly acknowledged and that the benefits of bioprinting are distributed equitably [78].

To address these challenges, the bioprinting community must adopt IP frameworks that balance innovation incentives with ethical considerations and societal needs. This includes redefining ownership models for bioprinted tissues, incorporating patients’ rights into licensing agreements, and establishing international guidelines to harmonize IP protections. Patented biological structures present a complex issue in bioprinting, particularly when it comes to bioprinted organ structures that replicate patented designs or techniques. Patent law has traditionally protected inventions that are novel, non-obvious, and useful, and this framework extends to bioprinting technologies [79]. However, the application of these rules becomes more nuanced when considering biological constructs, such as tissues or organs, that might closely resemble naturally occurring structures or previous patented works.

One of the primary legal challenges in bioprinting involves determining whether a bioprinted organ or tissue can be considered a new invention or an infringement on existing patents [80]. For instance, if a researcher uses patented techniques to arrange cells or apply certain biomaterials to produce a bioprinted organ, questions arise regarding whether the resulting product is sufficiently novel to warrant its own patent or whether it falls under the scope of the original patent. If the bioprinted organ structure replicates a patented design or uses a patented method to create a certain tissue arrangement, this could lead to legal disputes over patent infringement.

The issue becomes particularly complex when the bioprinted organ incorporates aspects of existing biological structures that are already patented, such as specific cell arrangements or methods for scaffolding. For example, a patent holder might claim the proprietary right to a specific method of creating vascular structures within bioprinted organs [81]. If another bioprinter or researcher uses a similar method without permission, they could be infringing on the original patent. The challenge in these cases lies in determining whether the bioprinted organ involves an innovative enough modification to be exempt from infringement or whether it constitutes a derivative work that falls within the scope of the existing patent.

The legal landscape for patenting biological structures, such as organs and tissues, is still evolving. Courts will need to balance the goals of fostering innovation with the need to avoid unjust monopolies over fundamental biological processes. Further complicating matters is the concept of “natural products,” which patent law traditionally excludes [82]. As bioprinting becomes more sophisticated, it is likely that new legal precedents will emerge that address the intersection of patent law with the unique nature of bioprinted biological materials.

Liability and responsibility for intellectual property (IP) infringement in the context of bioprinted products can be equally complex. Determining who is responsible when a bioprinted organ or tissue infringes on a patent is an ongoing debate, and several parties may share in the liability [83]. Typically, patent infringement liability lies with the entity or individual that directly engages in the act of infringing, such as the designer of the bioprinted structure, the bioprinter manufacturer, or the healthcare provider using the bioprinted product. The designer or researcher who develops the bioprinted organ might be held liable if they knowingly use patented techniques or designs without permission. However, responsibility may also extend to the bioprinter manufacturer, particularly if the device is marketed or used in a way that facilitates infringement. For example, if the printer itself is designed to reproduce patented organ structures or uses proprietary software that enables the creation of patented designs, the printer manufacturer could be liable for patent infringement [84].

Healthcare providers using bioprinted tissues or organs may also face legal challenges. If a healthcare provider knowingly uses a bioprinted organ that infringes on a patent, they could be seen as complicit in the infringement, particularly if they are aware of the patent and continue to use the product [85]. However, in many cases, healthcare providers may not have the technical expertise to evaluate the underlying patents or products used in bioprinting. This raises questions about whether the burden of responsibility should fall on the provider or the manufacturer of the bioprinted products. Table 4 captures the intersection of legal, ethical, and commercial challenges, offering structured insights into addressing the IP complexities of bioprinted tissues and organs.

Legal frameworks addressing patent infringement in the context of bioprinting are still developing, and there is a need for clearer guidelines on the division of responsibility. As bioprinting technology continues to evolve, it will be critical to establish frameworks that clarify the roles and liabilities of different parties in the bioprinting process, ensuring fair use of innovations while protecting the rights of patent holders.

## 5. Regulatory Frameworks and Ethical Considerations

The development of bioprinting technologies introduces numerous challenges related to intellectual property (IP) laws, particularly as they intersect with biotechnology and medical device regulations. Existing legal frameworks for biotech and medical devices, which have traditionally governed the patentability of biological innovations and the regulation of medical devices, are being tested by the emergence of bioprinting [86]. The applicability of these laws to bioprinting depends on how these technologies are classified, whether as medical devices, biotechnology, or a hybrid of both, and how they are used in various contexts, such as research, clinical settings, and commercial applications.

In terms of intellectual property, the existing framework for biotechnology generally provides protection for novel biological inventions, including genetically modified organisms, biopharmaceuticals, and biotech processes. Patent laws, including those under the U.S. Patent Act and European Patent Convention, typically grant protection to novel biotechnological processes, products, and materials if they meet the criteria of novelty, non-obviousness, and utility [87]. Bioprinting, particularly the use of 3D printing technologies to create complex tissue structures, can be covered by these frameworks if the printed tissue or organ involves an innovative process or material composition. However, challenges arise in determining the patentability of the tissues themselves, as natural biological structures are often excluded from patent protection unless they are significantly modified or enhanced through human intervention. This issue is particularly pronounced in the case of human tissues and organs, which, while novel in their bioprinted form, might still be argued to be “products of nature” and thus outside the scope of traditional patent protection.

Bioprinting technologies also intersect with medical device regulations, especially in the context of bioprinted products intended for clinical use. Medical devices are regulated by agencies like the U.S. Food and Drug Administration (FDA) or the European Medicines Agency (EMA), which require rigorous testing and validation to ensure safety and efficacy. Bioprinted products that are used for therapeutic or diagnostic purposes are likely to be subject to these medical device regulations [88,89]. For instance, bioprinted scaffolds or tissues used in regenerative medicine would be classified as medical devices and subject to regulatory oversight under frameworks such as the FDA’s 21st Century Cures Act [90]. However, given the rapid pace of technological advancements in bioprinting, current regulations may not fully address the complexities of these innovations, necessitating the development of new guidelines that explicitly account for the unique characteristics of bioprinted tissues and organs.

On an international level, IP protection for bioprinting technologies varies significantly across countries. While countries like the United States and European Union members have relatively established patent frameworks for biotechnology, other jurisdictions are still developing their approaches. For example, the United States has a robust framework for protecting biotechnology-related innovations, including bioprinted products, under the U.S. Patent and Trademark Office (USPTO) [91]. The European Union also offers similar protections, though there is an ongoing debate in Europe about the patentability of bioprinted human tissues, especially those created for medical applications. In contrast, countries with less established IP laws for biotechnology, such as some emerging economies, may offer weaker protections for bioprinting innovations, creating challenges for multinational collaborations and commercial ventures.

Furthermore, cross-disciplinary perspectives add additional layers of complexity. For instance, the intersection of biotechnology, medical device regulations, and digital technologies like 3D printing requires an interdisciplinary approach to IP protection. The bioprinting field involves contributions from fields such as materials science, bioengineering, and computer-aided design (CAD), each of which has its own IP protocols. This complexity is further exacerbated when considering the application of bioprinted products in healthcare, where patient rights, public health considerations, and access to innovative treatments must be balanced against the protection of commercial interests. In countries with strong IP protections, bioprinting innovations are likely to face fewer barriers to commercialization, but in regions where IP enforcement is weaker, the risks of patent infringement and misappropriation of bioprinted technologies are higher. An essential aspect of addressing cross-border IP challenges in bioprinting lies in the harmonization of regulatory frameworks across jurisdictions with varying levels of technological and economic development. While international collaboration is often highlighted as a solution, significant practical barriers must be acknowledged. For instance, conflicting national laws, disparities in technological infrastructure, and eco-nomic inequalities can hinder the effective implementation of unified frameworks. In particular, researchers in developing countries face unique challenges, including limited access to advanced bioprinting technologies, restrictive funding environments, and reduced participation in global policymaking efforts. Addressing these disparities will require targeted measures, such as capacity-building initiatives, equitable access to shared resources, and international agreements that consider the diverse needs of stakeholders across the global scientific community.

Intellectual property (IP) protection for bioprinting technology varies significantly across regions, with distinct challenges and approaches in countries across Asia and Africa. In Asia, countries like Japan and South Korea have robust IP frameworks that integrate biotechnological innovations, including bioprinting [92]. Japan, for instance, has established specific patent protections for bioprinting technologies such as bioinks and 3D printing hardware, promoting innovation while ensuring the protection of proprietary technologies [93]. Similarly, South Korea’s IP laws support bioprinting by allowing the patenting of genetically modified tissues and organs, though there is ongoing debate regarding the ethical implications of patenting biological materials [94]. In contrast, many African countries face a more fragmented approach to IP protection for bioprinting. Limited legal infrastructure and resources often result in inconsistent enforcement of IP rights, posing significant challenges for innovators. Countries such as South Africa are making strides by adapting their patent laws to account for biotechnological developments, but many African nations still lack comprehensive regulations specifically addressing bioprinting [95,96]. This disparity underscores the need for greater international collaboration and the development of region-specific IP frameworks to foster innovation and protect bioprinting advancements globally.

One of the primary ethical dilemmas is the potential for cloning. The ability to print tissues and organs that closely replicate the biological structures of a human body, particularly using stem cells or genetic material, sparks debates over whether this constitutes a form of human cloning. While bioprinting in its current form does not involve the creation of fully cloned humans, the possibility of replicating tissues that are genetically identical to an individual’s own raises concerns about identity and personhood. If bioprinted organs or tissues are created from a person’s own cells, questions emerge about the potential for creating duplicate biological material that could be used for unintended purposes, including identity theft, reproduction, or unauthorized enhancements. The ethical debate on cloning extends to the concern of whether bioprinting could be a stepping stone to reproductive cloning or the creation of genetically modified embryos.

Human enhancement is another significant ethical issue, where bioprinting may blur the lines between medical treatment and enhancement. If bioprinted organs are used not just to replace damaged tissues but to enhance human capabilities beyond their natural state, ethical concerns arise about the potential for social inequality. For example, wealthy individuals may be able to afford enhanced or optimized bioprinted organs, leading to a divide between those who can access advanced bioprinting treatments and those who cannot. Moreover, bioprinted enhancements could lead to the pursuit of “designer” organs or tissues that enhance physical attributes, cognitive functions, or longevity. This brings up concerns about the possible commercialization of human biology and the exacerbation of social inequalities, where only a privileged few benefit from these technologies, potentially leading to a society where access to biological enhancements becomes a marker of socio-economic status.

To address these complex ethical dilemmas, several proposed solutions and innovations have been discussed. One of the key frameworks for addressing these concerns is the development of specific bioprinting regulations that balance technological advancements with ethical considerations [97]. Many bioethicists and legal scholars argue that bioprinting should be tightly regulated to ensure that it is used primarily for therapeutic, rather than enhancement, purposes. Regulations could mandate that bioprinted organs or tissues be used exclusively for medical needs, such as treating diseases or replacing damaged organs, and explicitly prohibit the use of bioprinting for enhancement or cosmetic procedures [98]. Additionally, a clear distinction should be made between the creation of bioprinted organs for therapeutic purposes and the creation of genetically modified embryos or organs for reproductive cloning.

Another important consideration is the establishment of industry standards for bioprinting technologies [99]. These standards could help prevent exploitation and ensure that the technology is used in ways that are ethically and socially responsible. For example, establishing protocols for the sourcing and use of biological materials—such as ensuring that human tissues and cells are obtained with informed consent—could safeguard against unethical practices. Furthermore, industry standards could help ensure that bioprinted organs are accessible to all, not just the affluent, by creating a framework for equitable distribution based on need rather than financial ability [100]. Table 5, discuses the regulatory frameworks and ethical considerations in bioprinting.

Finally, international collaboration on bioethical guidelines and cross-border regulations is essential given the global nature of the bioprinting industry. Different countries have varying standards for medical ethics and IP protections, which can create inconsistencies in how bioprinted products are regulated and used. To address this, international treaties or agreements could help create a unified ethical framework for bioprinting, ensuring that these technologies are used in ways that respect human dignity, equity, and fairness across different jurisdictions.

## 6. Conclusions and Future Directions

Bioprinting represents a transformative intersection of additive manufacturing and biotechnology, with significant potential to revolutionize healthcare, pharmaceuticals, and regenerative medicine. However, the integration of intellectual property (IP) frameworks into this rapidly evolving field exposes critical legal, ethical, and technical challenges that demand practical and actionable solutions. Traditional IP systems, while foundational, are inadequate to address the unique complexities of bioprinting, particularly those related to the ownership and patentability of bioinks, bioprinting hardware, and digital design files derived from biological data. Furthermore, the commodification of bioprinted tissues and organs raises profound ethical dilemmas that complicate the establishment of comprehensive regulatory frameworks.

To address these challenges, future efforts must prioritize the development of hybrid IP systems that balance open innovation with mechanisms for safeguarding proprietary technologies. For instance, the successful implementation of tailored licensing frameworks in related fields, such as biotechnology, offers a useful model for fostering collaborative innovation while ensuring adequate protection for contributors. Such frameworks should draw from real-world case studies to establish best practices for harmonizing open access with proprietary rights. Moreover, international legal harmonization is essential to mitigate jurisdictional disparities and enable the equitable development and regulation of bioprinting technologies. A comparative analysis of existing global IP frameworks can provide valuable insights into the practical steps required to achieve such harmonization.

Ethical oversight must also become a cornerstone of the IP landscape in bioprinting. Independent review boards, modeled after those in clinical research, could play a critical role in ensuring the responsible use of biological materials and adherence to principles of equity and transparency. Drawing on examples from fields such as genomics, the integration of dynamic consent models could provide a flexible and ethical approach to managing patient-derived biological data, safeguarding privacy while enabling innovation. Additionally, addressing implementation challenges, such as conflicting regulatory standards and economic disparities, is crucial for ensuring that ethical guidelines are not only conceptual but also actionable and impactful.

In conclusion, the progress of bioprinting depends on promoting interdisciplinary collaboration among legal, technical, and ethical experts to build an IP framework that addresses the identified challenges with concrete solutions. By explicitly aligning recommendations with real-world precedents and implementation strategies, stakeholders can develop an IP system that supports innovation while addressing ethical and societal concerns. Proactively addressing these challenges will enable bioprinting to realize its full potential in the area of personalized medicine, drug development, and organ transplantation, ensuring that its benefits are distributed sustainably and equitably across global communities.

## Figures and Tables

**Table 1:** Key challenges, legal complexities, and proposed solutions for intellectual property in the general bioprinting domain.

IP Area	Key Challenges	Legal Complexities	Potential Frameworks
**Bioprinting Hardware and Materials**	- Patentability of novel 3D printing machines and bioprinting materials (bioinks, scaffolds)	- Determining novelty and inventiveness in hardware and material design; conflicting patent claims	- Clear guidelines for patenting bioprinting machines and materials; public-private collaborations to define patent scope
**Digital Design Files**	- Copyrightability and ownership of design files; data privacy concerns	- Protection of digital blueprints based on biological data; infringement risks with open-source files	- Copyright protection for digital designs; development of secure data-sharing platforms to safeguard digital assets
**Bioprinted Products**	- Ownership and patentability of bioprinted tissues, organs, and customized products; commercial rights	- Ethical considerations regarding the patenting of human cells and tissues; regulatory hurdles in product approval	- Ethical IP policies addressing living organisms; international cooperation on regulation and commercialization processes
**Collaboration and Multidisciplinary Innovation**	- Defining ownership in multi-disciplinary teams working on bioprinting projects; joint inventions	- Disputes over contributions and inventorship; unclear rules on joint patent filings	- Development of clear IP agreements in collaborative projects; joint patenting models for bioprinting innovations
**Ethical and Regulatory Concerns**	- Ethical dilemmas around patenting biological materials and processes	- Balancing IP rights with the ethical implications of manipulating living organisms; regulatory compliance	- Creation of ethical review boards to guide IP decisions; integration of ethical guidelines into bioprinting patent law

**Table 2:** Key challenges, legal complexities, and proposed solutions for intellectual property in bioprinting hardware and materials.

Category	Key Challenges	Legal Complexities	Proposed Solutions
**Bioprinting Hardware**	Patentability of novel 3D bioprinters and components	Incremental innovations may overlap with existing patents; interdisciplinary contributions blur ownership	Clear guidelines for defining novelty and inventive steps; joint patent agreements in multidisciplinary projects
	Lack of standards for proprietary mechanisms	Disputes over claims for precision, multi-material deposition, or environmental controls	International collaboration to establish hardware standards and patent scope
**Bioinks**	Ownership and patentability of bioinks containing living cells and biomaterials	Ethical concerns over human-derived materials; challenges proving novelty and utility	Ethical IP policies; frameworks allowing limited-term exclusivity while ensuring fair use for medical purposes
	Proprietary formulations and trade secrets for bioink development	Risk of misappropriation due to insufficient IP protections	Enhanced confidentiality agreements; hybrid models of patents and trade secrets
**Support Materials**	IP disputes over biodegradable or innovative scaffolding materials	Balancing proprietary rights with open-access frameworks for further research	Licensing agreements encouraging collaborative innovation
**Process Innovations**	Patentability of unique bioprinting techniques	Overlapping claims for methods used in cell placement or bioink deposition	Detailed patent claims delineating methods and applications

**Table 3:** Intellectual property challenges, legal complexities, and proposed solutions for digital files and biological data in bioprinting.

Category	Key Challenges	Legal Complexities	Proposed Solutions
**Digital Design Files**	Ownership and copyright of bio-CAD files	Difficulty distinguishing between creative designs and functional aspects	IP frameworks distinguishing artistic content (copyright) from technical processes (patents)
	Risks of unauthorized replication and distribution of design files	Open-source sharing increases potential for infringement	Development of secure sharing platforms; licensing models tailored for digital design files
**Patient-Derived Data**	Ownership and use of patient-specific data for digital modeling	Disputes over data ownership between patients, healthcare providers, and companies	Dynamic consent models; clear agreements delineating ownership and use rights
	Privacy concerns over genetic and anatomical data	Compliance with privacy laws (e.g., GDPR, HIPAA); cross-border data transfer risks	Robust anonymization protocols; international harmonization of data privacy standards
**Regulatory Compliance**	Ethical use of sensitive biological data	Lack of standardized guidelines for handling patient-derived data	Creation of ethical oversight boards to ensure compliance and patient autonomy
**Commercialization of Models**	Licensing and commercialization of bio-CAD files and patient-derived models	Balancing IP protections with equitable access	Tiered licensing frameworks that differentiate between research and commercial applications

**Table 4:** Key challenges, legal complexities, and proposed solutions for intellectual property in bioprinted tissues and organ replication.

Category	Key Challenges	Legal Complexities	Proposed Solutions
**Ownership of Bioprinted Products**	Determining ownership of tissues and organs derived from human cells	Ethical concerns about commodifying living tissues; unclear regulations around ownership	Development of ethical IP frameworks balancing innovation and equitable access
	Disputes over ownership rights when multiple contributors are involved	Multi-stakeholder claims complicate patent filings	Joint ownership agreements and clear attribution policies in collaborative projects
**Patentability**	Patent eligibility of bioprinted tissues/organs	Biological components challenge traditional patent criteria (novelty, utility, non-obviousness)	Revising patent laws to include living organisms and biological processes under specific ethical guidelines
	Ethical opposition to patenting human-derived products	Concerns over limiting access to life-saving technologies	Implementing limited-term exclusivity for commercial use while ensuring fair access
**Regulatory Approval**	Overcoming regulatory hurdles for commercialization	Lack of global standardization for approving bioprinted medical products	Harmonization of international regulations to facilitate market entry
**Commercialization**	Balancing profit-driven commercialization with equitable healthcare access	Risk of creating inequities in access to bioprinted tissues and organs	Tiered pricing models and non-commercial licensing options for underserved regions
**Ethical Oversight**	Addressing moral implications of bioprinting living tissues	Lack of consensus on ethical guidelines for bioprinted organ use	Establishment of international ethical review boards for bioprinting research and commercialization

**Table 5:** Regulatory frameworks and ethical considerations in bioprinting.

Aspect	Description	Key Considerations
**Regulatory Frameworks**	Governmental and international laws overseeing bioprinting.	Compliance with health and safety standards, regulations for medical use, and approval processes for bioprinted organs or tissues.
**Intellectual Property**	Copyright, patents, and trademarks related to bioprinted materials.	Patenting bioprinting technology, ownership of 3D printing files, and patenting bioprinted products.
**Ethical Issues**	Moral concerns surrounding the use of bioprinting in human tissues and organs.	Ensuring patient consent, avoiding misuse of bioprinting for unethical purposes, and addressing potential social inequality in access to bioprinted treatments.
**Global Approaches**	International efforts to standardize bioprinting practices.	Collaboration between countries for shared regulation and ethical standards to prevent exploitation of bioprinting technologies.
**Commercialization**	The transition of bioprinting technology from research to market.	Evaluating the risks of commercialization on safety, accessibility, and the regulation of bioprinted medical devices and implants.

## Data Availability

Not applicable.
